# COVID-19 assessment in family practice—A clinical decision rule based on self-rated symptoms and contact history

**DOI:** 10.1038/s41533-021-00258-4

**Published:** 2021-11-25

**Authors:** Antonius Schneider, Katharina Rauscher, Christina Kellerer, Klaus Linde, Frederike Kneissl, Alexander Hapfelmeier

**Affiliations:** 1 TUM School of Medicine, Institute of General Practice and Health Services Research, Technical University of Munich, Munich, Germany; 2 TUM School of Medicine, Institute of Medical Informatics, Statistics and Epidemiology, Technical University Munich, Munich, Germany

**Keywords:** Respiratory signs and symptoms, Epidemiology

## Abstract

The study aimed to evaluate the diagnostic accuracy of contact history and clinical symptoms and to develop decision rules for ruling-in and ruling-out SARS-CoV-2 infection in family practice. We performed a prospective diagnostic study. Consecutive inclusion of patients coming for COVID-PCR testing to 19 general practices. Contact history and self-reported symptoms served as index test. PCR testing of nasopharyngeal swabs served as reference standard. Complete data were available from 1141 patients, 605 (53.0%) female, average age 42.2 years, 182 (16.0%) COVID-PCR positive. Multivariable logistic regression showed highest odds ratios (ORs) for “contact with infected person” (OR 9.22, 95% CI 5.61–15.41), anosmia/ageusia (8.79, 4.89–15.95), fever (4.25, 2.56–7.09), and “sudden disease onset” (2.52, 1.55–4.14). Patients with “contact with infected person” or “anosmia/ageusia” with or without self-reported “fever” had a high probability of COVID infection up to 84.8%. Negative response to the four items “contact with infected person, anosmia/ageusia, fever, sudden disease onset” showed a negative predictive value (NPV) of 0.98 (95% CI 0.96–0.99). This was present in 446 (39.1%) patients. NPV of “completely asymptomatic,” “no contact,” “no risk area” was 1.0 (0.96–1.0). This was present in 84 (7.4%) patients. To conclude, the combination of four key items allowed exclusion of SARS-CoV-2 infection with high certainty. With the goal of 100% exclusion of SARS-CoV-2 infection to prevent the spread of SARS-CoV-2 to the population level, COVID-PCR testing could be saved only for patients with negative response in all items. The decision rule might also help for ruling-in SARS-CoV-2 infection in terms of rapid assessment of infection risk.

## Introduction

Rapid and accurate assessment of patients with suspected severe acute respiratory syndrome coronavirus type 2 (SARS-CoV-2) infection upon presentation to the primary care practices is of paramount importance to ensure optimal diagnostic and therapeutic management. Infected patients must be reliably identified in order to initiate quarantine measures and organize appropriate patient care. In addition, it is important not to misclassify uninfected individuals as infected, thereby frightening them and excluding them from work and social life. Efficient testing strategies are valuable not only to ensure accurate patient assessment but also to prevent the spread of SARS-CoV-2 at the population level. Therefore, a low-threshold testing strategy has been implemented in many countries, also in Germany^1,2^. Since early summer 2020, patients in Germany were allowed to receive a polymerase chain reaction (PCR) nasopharyngeal swab in their family physicians’ office if they had symptoms, felt uncertain about a possible infection, or needed a test result for legal reasons. Thus, access to testing was relatively easy, without selection of patients. This testing strategy has resulted in a large volume of COVID-PCR, which is time-consuming and costly.

Medical history and clinical signs and symptoms might contribute to an efficient selection of patients, in particular when decision rules can be developed to facilitate diagnostic decision making regarding test ordering of corona virus disease 2019 (COVID)-PCR. A systematic review including all studies from primary care and hospital settings has shown that most clinical signs and symptoms have high specificity but low sensitivity, thus making it difficult to exclude the disease^3^. Only four studies have been conducted in the primary care setting^4–7^. Anosmia^4–7^, fever^5^, and first grade contact with an infected person^4^ was found to be predictive for SARS-CoV-2 infection. However, a combination of signs and symptoms were not evaluated in these studies, while the urgent need for prospective studies in an unselected population presenting to primary care has been identified^3^. The aim of the present study was to evaluate the diagnostic accuracy of anamnesis, contact history and clinical symptoms and to develop medical decision rules for ruling-in and ruling-out of SARS-CoV-2 infection in family practice.

## Methods

### Study design

This prospective diagnostic study was conducted from November 25, 2020 to February 26, 2021 in nineteen family practices in urban and rural areas of Upper Bavaria, Germany. The practices are part of a network that includes a total of 210 teaching practices at the Institute which are average practices representative for all practices in that area. All participating practices provide unselected primary care in the community for people making an initial approach to a medical professional within the social health insurance system. All patients (at least 18 years old) who came for COVID-PCR were asked to complete a short questionnaire on medical history, self-reported symptoms (SRS) and possible contact with (potentially) infected persons. Patients were included consecutively. The questionnaire items were generated from literature research and from information about the core symptoms of the disease as outlined by the website of the Robert Koch Institute^8^. The Robert Koch Institute (RKI) is the government’s central scientific institution in the field of biomedicine. Anamnestic items, contact history and SRS served as an index test (for queried symptoms, see Table 1). Nasopharyngeal swabs were performed and sent to the local medical laboratory for PCR analysis (reference standard). In Germany, the diagnostic decision is based on absolute detection of viral RNA in PCR analysis using a cycle threshold (Ct) of at least 40 cycles. The study was approved by the Ethics Committee of the Medical Faculty of the Technical University of Munich. All patients received written study information, and informed consent was obtained from all participants.

**Table 1:** Multivariable regression model of the questionnaire items.

Questionnaire item	Multivariable regression OR	*p* value
Demographics		
(1) Sex = male	1.17 (0.79, 1.73)	0.436
(2) Age (in years)	1.03 (1.01, 1.05)	<0.001
Symptoms		
(3) Anosmia/ageusia	8.79 (4.89, 15.95)	<0.001
(4) Fever—yes	4.25 (2.56, 7.09)	<0.001
5) Sudden disease onset	2.52 (1.55, 4.14)	<0.001
(6) Limp pain	1.72 (1.02, 2.91)	0.041
(7) Dry cough	1.69 (1.08, 2.62)	0.020
(8) Headache	1.14 (0.70, 1.84)	0.598
(9) Common cold	1.05 (0.67, 1.65)	0.822
(10) Fatigue	1.01 (0.58, 1.75)	0.978
(11) Diarrhea	0.91 (0.49, 1.61)	0.742
(12) Sore throat	0.52 (0.32, 0.83)	0.006
(13) Dyspnea	0.32 (0.14, 0.69)	0.005
Medical history		
(14) Nicotin use	0.45 (0.25, 0.76)	0.004
(15) Chronic disease	0.34 (0.20, 0.57)	<0.001
Contact history		
(16) Contact with infected person	9.22 (5.61, 15.41)	<0.001
(17) Stay in corona risk area	1.48 (0.67, 3.08)	0.310
(18) Contact with persons with suspected infection	1.28 (0.78, 2.07)	0.324

Variables in subcategories are ordered according to odds ratios (OR) of multivariable regression analysis (*n* = 1141). Intercept of the logistic regression model: −4.653

### Statistical analysis

The distribution of continuous data is described by means and standard deviations. Qualitative data are presented by absolute and relative frequencies. Descriptive data were analyzed with *t* test or Chi-Square-test. Sensitivities, specificities, positive predictive values (PPV), negative predictive values (NPV), positive likelihood ratios, negative likelihood ratios, diagnostic odds ratios (ORs), and respective 95% confidence intervals (CIs) were computed for the items of the questionnaire. In some countries, high-risk contacts are dealt with in a separate testing strategy than symptomatic patients. Therefore, sensitivity analyses were performed without the “contact history” variables.

Investigated statistical models and machine learning methods were a conditional inference decision tree, a respective random forest, a Lasso model with the best cross-validated performance, a sparser Lasso model that did not perform significantly worse (using the 1se rule), and two multivariable logistic regression models built with and without Akaike Information Criterion-based stepwise backward variable selection^9^. The performance of diagnostic modeling was measured by the area under the receiver operating characteristics curve (AUC). A benchmark study was conducted to compare and internally validate the models’ performance by fivefold cross-validation. Thus, each model was repeatedly built on parts of the data (i.e., training data) and applied to independent parts of the data (i.e., test data) for an unbiased internal performance evaluation. For effect estimation and interpretation purposes, another conditional inference decision tree and a multivariable logistic regression model were refit to the whole data. ORs with 95% CIs are presented for the latter. Only patients with complete data were analyzed. For sample size calculation, we applied the rule of thumb 1:10 for the ratio of the model parameters to the number of observations in the less frequent outcome class^10^. Therefore, limiting sample size of 180 test positives had been determined a priori to allow consistent effect estimation of 18 model parameters in a multivariable logistic regression model^10^. More advanced rules, e.g., according to Riley et al.^11^, involving the anticipated outcome proportion and model performance have not been applied due to the dynamic development of the pandemic, which did not allow reliable assumptions to be made a priori^11^. Significance of group differences and regression coefficients were assessed at exploratory two-sided alpha levels of 5%. Computations were conducted with R 4.0.3 (The R Foundation for Statistical Computing, Vienna, Austria).

As the PCR results were available to us on a daily basis, we were able to enroll patients into the study until the predefined sample size was reached. The data were entered twice by K.R. A comparison was made by inspection; in the event of a mismatch of variables, the information in the original questionnaire was checked and adopted in the data set. The statistical analysis was performed by the statistician A.H.

### Reporting summary

Further information on research design is available in the Nature Research Reporting Summary linked to this article.

## Results

### Baseline characteristics

**Fig. 1:**
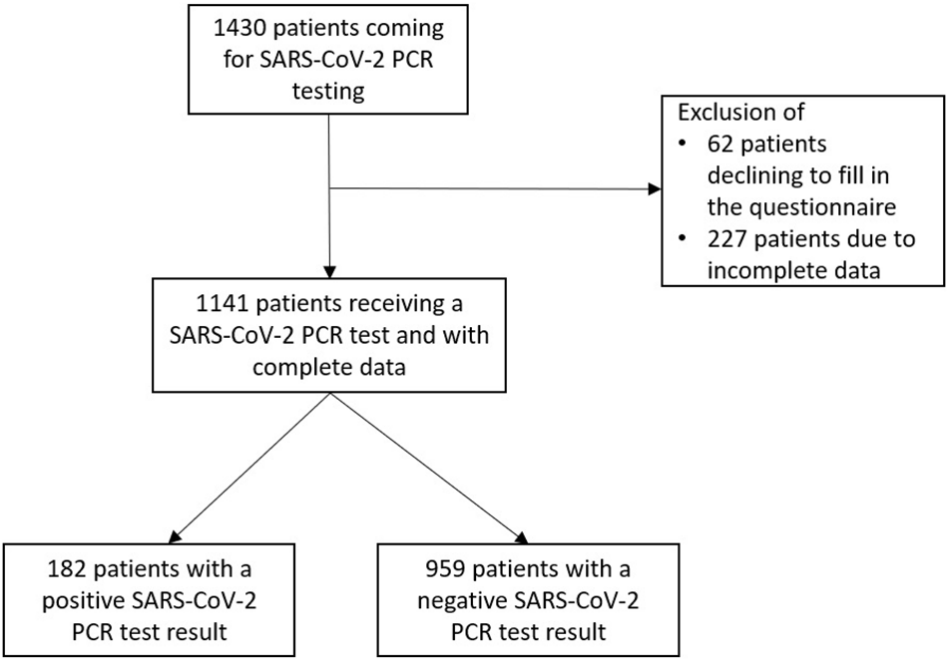
Flow chart of the patients. Consecutive recruitment of patients in 19 general practices, depicted by exclusion criteria and PCR test results.

1430 patients coming for PCR testing were invited, and 1368 (95.7%) patients participated. Of these, 707 (51.7%) patients were female, average age was 42.5 (SD = 16.4) years. 214 (15.0%) patients had a positive PCR test result. Data of 1141 (79.8%) patients who completely filled in their questionnaires were available for analysis and diagnostic modeling (Fig. 1). Of these, 605 (53.0%) patients were female, average age was 42.2 (SD = 16.4). 182 (16.0%) had a positive PCR test result. Patients with incomplete questionnaires were 3.1 years older (*p* = 0.030) and complained more often about dry cough (42,1%; *p* = 0.013) or headache (47,7%; *p* = 0.057). Sixty-two patients declined to fill in the questionnaire, 37 (59.7%) were female and the average age was 46.4 (SD = 20.0) years.

### Analysis of the AUCs

**Fig. 2:**
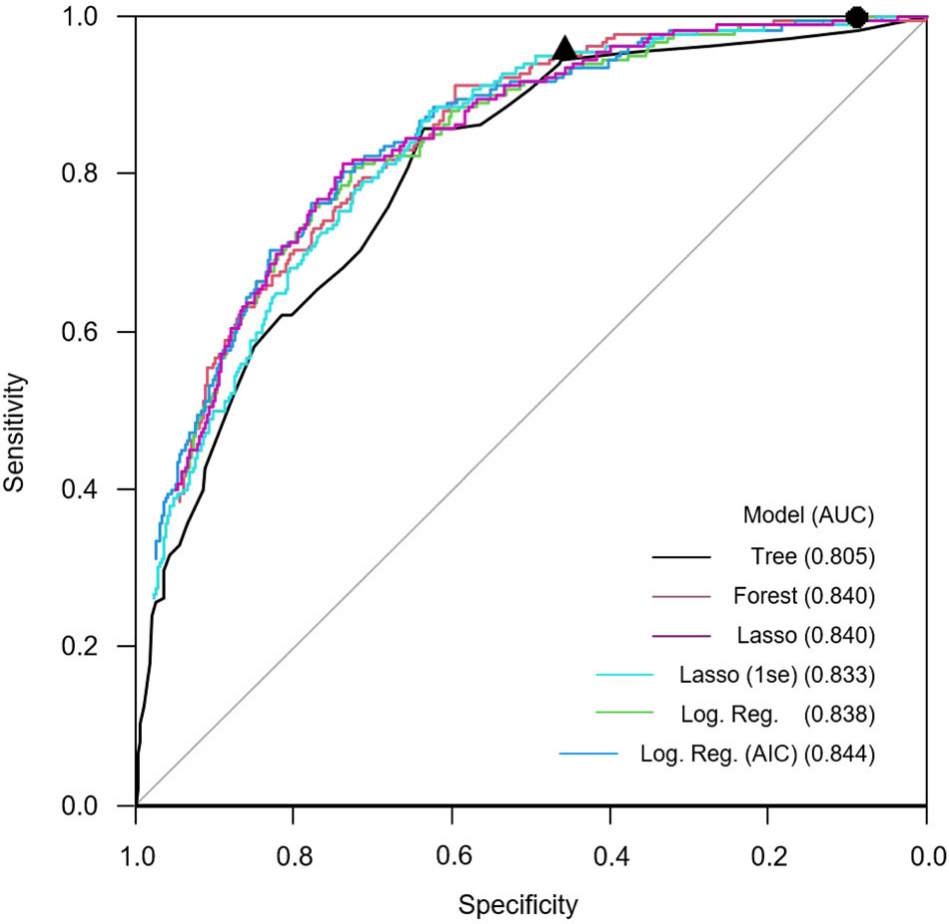
ROC curves and AUC of the listed multivariable diagnostic models predicting a SARS-CoV-2-positive PCR test result. All questionnaire items (see Table 1), including age and sex, were offered to the algorithms. The sensitivity and specificity of specific decision rules are added as individual points. Black circle (●) represents at least one symptom #3–18 and/or contact with (suspected) infected patient (see Table 1). Black triangle (▴) represents at least one positive response to the four items: contact with infected person, anosmia/ageusia, fever, and sudden disease onset.

The AUCs of the various models was similar and ranging from 0.805 to 0.844, which is estimated as an excellent diagnostic performance overall (Fig. 2)^12^. Multivariable logistic regression showed highest ORs for “contact with infected person” (OR 9.22, 95% CI 5.61–15.41), “anosmia/ageusia” (8.79, 4.89–15.95), “fever” (4.25, 2.56–7.09), and “sudden disease onset” (2.52, 1.55–4.14) (Table 1). “Dry cough” (1.69, 1.08–2.62) and “limb pain” (1.72, 1.02–2.91) showed a weaker association with SARS-CoV-2 infection. “Sore throat” (0.52, 0.32–0.83), “dyspnea” (0.32, 0.20–0.57), “nicotine use” (0.45, 0.25–0.76), and “chronic disease (0.34, 0.20–0.57) were associated with a reduced risk for SARS-CoV-2 infection. Risk of infection increased with increasing age (1.03, 1.01–1.05). The specificities of all questionnaire items were higher than the sensitivities (Table 2). All NPVs were >80%, whereas the PPVs were comparatively low. The highest PPV (0.45, 0.35–0.55) was given in anosmia/ageusia.

**Table 2:** Diagnostic measures of the questionnaire items and selected decision rules.

	Sensitivity	Specificity	Predictive values		Likelihood ratio		Diagnostic OR
			Positive	Negative	Positive	Negative	
Demographics							
(1) Sex = male	0.48 (0.41, 0.56)	0.53 (0.50, 0.56)	0.16 (0.13, 0.20)	0.84 (0.81, 0.87)	1.04 (0.88, 1.22)	0.97 (0.83, 1.13)	1.07 (0.78, 1.47)
(2) Age >39 years	0.60 (0.52, 0.67)	0.51 (0.48, 0.54)	0.19 (0.16, 0.22)	0.87 (0.84, 0.90)	1.22 (1.07, 1.40)	0.79 (0.65, 0.95)	1.56 (1.13, 2.15)
Symptoms							
(3) Anosmia/ageusia	0.25 (0.19, 0.32)	0.94 (0.92, 0.96)	0.45 (0.35, 0.55)	0.87 (0.85, 0.89)	4.33 (3.03, 6.18)	0.79 (0.73, 0.86)	5.45 (3.55, 8.38)
(4) Fever—yes	0.34 (0.27, 0.41)	0.87 (0.84, 0.89)	0.33 (0.26, 0.40)	0.87 (0.85, 0.89)	2.55 (1.97, 3.31)	0.76 (0.68, 0.85)	3.35 (2.34, 4.80)
(5) Sudden disease onset	0.46 (0.39, 0.54)	0.71 (0.68, 0.74)	0.23 (0.19, 0.28)	0.87 (0.85, 0.90)	1.59 (1.32, 1.92)	0.76 (0.66, 0.87)	2.10 (1.52, 2.90)
(6) Limp pain	0.46 (0.39, 0.54)	0.72 (0.69, 0.75)	0.24 (0.20, 0.29)	0.88 (0.85, 0.90)	1.66 (1.38, 2.01)	0.75 (0.65, 0.86)	2.23 (1.62, 3.09)
(7) Dry cough	0.43 (0.36, 0.51)	0.69 (0.65, 0.71)	0.21 (0.17, 0.25)	0.86 (0.84, 0.89)	1.38 (1.14, 1.67)	0.83 (0.72, 0.94)	1.67 (1.21, 2.31)
(8) Headache	0.48 (0.41, 0.56)	0.61 (0.58, 0.64)	0.19 (0.15, 0.23)	0.86 (0.83, 0.89)	1.23 (1.04, 1.46)	0.85 (0.73, 0.99)	1.45 (1.05, 1.99)
(9) Common cold	0.43 (0.36, 0.50)	0.62 (0.59, 0.65)	0.18 (0.14, 0.22)	0.85 (0.82, 0.88)	1.14 (0.94, 1.37)	0.92 (0.80, 1.05)	1.24 (0.90, 1.71)
(10) Fatigue	0.54 (0.46, 0.61)	0.56 (0.53, 0.59)	0.19 (0.16, 0.23)	0.87 (0.84, 0.89)	1.23 (1.06, 1.44)	0.82 (0.69, 0.97)	1.50 (1.09, 2.07)
(11) Diarrhea	0.14 (0.10, 0.20)	0.85 (0.83, 0.87)	0.15 (0.10, 0.22)	0.84 (0.82, 0.86)	0.96 (0.66, 1.42)	1.01 (0.94, 1.07)	0.96 (0.61, 1.51)
(12) Sore throat	0.31 (0.25, 0.39)	0.60 (0.57, 0.63)	0.13 (0.10, 0.16)	0.82 (0.79, 0.85)	0.79 (0.63, 0.99)	1.14 (1.02, 1.28)	0.69 (0.49, 0.97)
(13) Dyspnea	0.07 (0.04, 0.12)	0.91 (0.88, 0.92)	0.12 (0.07, 0.20)	0.84 (0.81, 0.86)	0.75 (0.43, 1.32)	1.03 (0.98, 1.07)	0.73 (0.40, 1.34)
Medical history							
(14) Nicotin use	0.15 (0.10, 0.21)	0.73 (0.70, 0.76)	0.09 (0.06, 0.13)	0.82 (0.79, 0.84)	0.55 (0.38, 0.79)	1.17 (1.08, 1.25)	0.47 (0.31, 0.73)
(15) Chronic disease	0.17 (0.12, 0.23)	0.72 (0.69, 0.75)	0.10 (0.07, 0.14)	0.82 (0.79, 0.85)	0.61 (0.44, 0.86)	1.15 (1.06, 1.24)	0.53 (0.35, 0.80)
Contact history							
(16) Contact with infected person	0.58 (0.51, 0.65)	0.80 (0.77, 0.82)	0.35 (0.30, 0.41)	0.91 (0.89, 0.93)	2.86 (2.40, 3.41)	0.52 (0.44, 0.62)	5.46 (3.91, 7.63)
(17) Stay in corona risk area	0.08 (0.05, 0.13)	0.92 (0.90, 0.94)	0.17 (0.10, 0.26)	0.84 (0.82, 0.86)	1.07 (0.63, 1.82)	0.99 (0.95, 1.04)	1.07 (0.60, 1.92)
(18) Contact with persons with suspected infection	0.38 (0.31, 0.46)	0.83 (0.81, 0.86)	0.30 (0.25, 0.37)	0.88 (0.85, 0.90)	2.31 (1.83, 2.91)	0.74 (0.66, 0.83)	3.12 (2.21, 4.40)
Combinations							
At least one symptom or condition #3–18	1.00 (0.98, 1.00)	0.09 (0.07, 0.10)	0.17 (0.15, 0.20)	1.00 (0.96, 1.00)	1.10 (1.07, 1.12)	0.00 (0.00, -)	–
At least one positive response to “contact with infected person”, anosmia/ageusia, fever, sudden disease onset	0.96 (0.92, 0.98)	0.46 (0.42, 0.49)	0.25 (0.22, 0.28)	0.98 (0.96, 0.99)	1.76 (1.65, 1.88)	0.10 (0.05, 0.19)	18.29 (8.90, 37.57)
Combinations excluding “contact with infected person” (sensitivity analysis)							
At least one symptom or condition #17	0.91 (0.86, 0.95)	0.16 (0.14, 0.19)	0.17 (0.15, 0.20)	0.91 (0.85, 0.95)	1.09 (1.03, 1.15)	0.54 (0.33, 0.88)	2.03 (1.18, 3.49)
At least one positive response to anosmia/ageusia, fever, sudden disease onset	0.70 (0.63, 0.77)	0.62 (0.59, 0.66)	0.26 (0.22, 0.30)	0.92 (0.89, 0.94)	1.87 (1.65, 2.12)	0.48 (0.38, 0.60)	3.94 (2.80, 5.56)

### Decision tree modeling

**Fig. 3:**
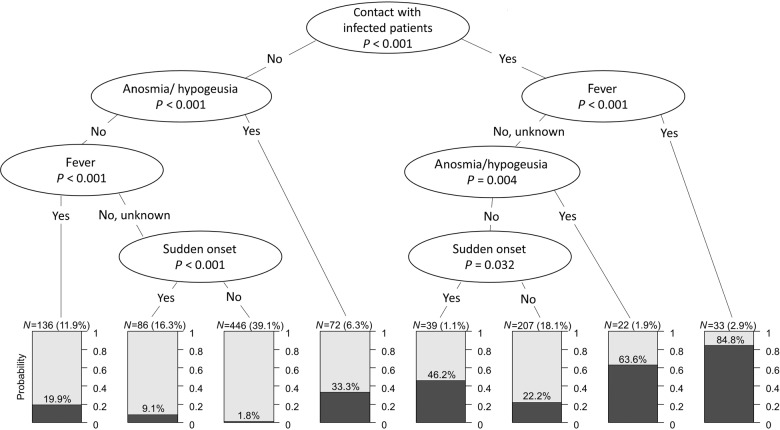
Conditional inference decision tree for the identification of a SARS-CoV-2-positive PCR test result. All questionnaire items (see Table 1), including age and sex, were offered to the algorithm.

The strongest predictors were also selected by the decision tree modeling procedure, strengthening the consistency of findings across models (Fig. 3). Patients with “contact with infected person” and/or “anosmia/ageusia” with or without self-reported “fever” had a high probability of SARS-CoV-2 infection up to 84.8%. SARS-CoV-2 infection could be ruled out with high certainty in patients with no “contact with infected person”, no “anosmia/ageusia”, no “fever,” and no “sudden disease onset”; the probability of SARS-CoV-2 infection was 1.8% in this group. The sensitivity of this combination was 0.96 (95% CI 0.92–0.98), and specificity was 0.46 (0.42–0.49), the negative predictive value (NPV) was 0.98 (0.96–0.99) (Table 2). This combination was present in 446 (39.1%) patients. The NPV of the decision rule “completely asymptomatic, no contact, no risk area” was 1.0 (0.96–1.0) (Table 2). This was existent in 84 (7.4%) patients. In the additional sensitivity analysis, we excluded the “contact history variables” from the calculation. The resulting PPV of “At least one positive response to anosmia/ageusia, fever, sudden disease onset” remained stable with PPV = 0.26, but the resulting NPV decreased to 0.92 (Table 2).

## Discussion

We found that the combinations of “contact with infected patients”, “anosmia/ageusia”, “fever,” and “sudden disease onset” might be particularly helpful for ruling-in and ruling-out SARS-CoV-2 infection. However, highest certainty for ruling-out with NPV = 100% is only given in completely asymptomatic patients without contact to an infected patient.

The association between clinical symptoms in our study were similar to previous studies in primary care^3–7^. However, the isolated symptoms were not strong enough to include or exclude SARS-CoV-2 infection. The combination according to our decision tree model revealed, that the negative response on the four key items allowed the exclusion of SARS-CoV-2 infection with NPV = 98%. This would also be true, when other symptoms such as “common cold” or “headache” are complained. These decision rules could be particularly helpful for clinical assessment of patients during consultations when the epidemic situation is tense or when the overall goal is mitigation. Depending on available resources, consideration could be given to omitting COVID-PCR testing in this group, which still represents more than one-third of the collective. However, this is not acceptable in terms of a COVID-19 elimination strategy^13^. With the goal of 100% exclusion of SARS-CoV-2 infection to prevent the spread of SARS-CoV-2 to the population level, COVID-PCR testing could only be saved in completely asymptomatic, “no contact”, “no risk area” patients, which would have been possible in 7.4% of the patient collective. Remarkably, SARS-CoV-2 infection could not be ruled-out satisfyingly without contact history information, because the resulting NPV = 0.92 would mean that 8% would be classified false negative.

The decision rule might also help for ruling-in SARS-CoV-2 infection. The probability of infection was higher than 80% in patients with previous contact with an infected person when fever is reported; and the presence of anosmia/ageusia was also highly predictive. In these cases, the medical treatment decision should ideally be made in a telephone consultation, possibly followed by a home visit, because these patients should not come into practice to prevent other patients and personnel from infection. In this context, the predictors could be used to make an initial triage decision during a phone call, possibly facilitated by implementation in practice software. Noteworthy, “dry cough” and “limb pain” were not relevant in the decision tree. This could be explained the fact that these symptoms were covered by the four key symptoms and thus did not provide any other additional diagnostic information. Beyond that, “nicotin use” and the presence of “chronic disease” was associated with a lower risk for SARS-CoV-2 infection. This might be explained by the fact that these patients are particularly aware of their risk of infection and therefore make special efforts to keep their distance and generally reduce the risk of infection as intensively as possible. “Sore throat” and “dyspnea” were already demonstrated to be associated with reduced risk for SARS-CoV-2 infection^7^, which is now replicated within our study. This may increase confidence that these symptoms are not indicative of SARS-CoV-2 infection.

A strength of the present study is the inclusion of nineteen practices for patient recruitment and the consecutive inclusion of a large patient collective with high willingness to participate. Regarding the background epidemic activity in Germany, the positive rate of PCR tests in the ambulatory practices in winter time 2020/21 was between 13.7 and 16.5%^14^, which corresponds to the positive rate in our study. These facts should allow a high generalizability of the results. In addition, the statistical analyses showed robust and consistent results. As a limitation, the predictive values found in our study must be interpreted in the context of the epidemic situation and the used test strategy. Yet, with a range of 87–307 daily new confirmed COVID-19 cases and 14,000–28,000 daily tests per million inhabitants and a low-threshold testing strategy^15^, the situation in Germany should be comparable with many other industrialized countries in winter 2020/2021. The data were collected in family practices in Upper Bavaria, Germany. The results might still be transferable due to the worldwide spread of COVID-19. However, it might be difficult to transfer the results to other settings such as nursing homes where elderly patients live, as they are more susceptible to SARS-CoV-2 infections. Likewise, our results may not be representative of children and young people who are often asymptomatic. Non-responders were slightly more female and elder than responders, with only a low risk of bias and not limiting the representativeness of our findings due to their small number. Beyond that, we could not analyze all data due to incomplete information in 227 (16.6%) patients. These patients were on average about 3 years older and complained more often about dry cough or headache, which might have affected their ability to completely fill in the questionnaire. The risk of bias that arises from the complete case analysis was therefore considered to be low. In addition, most of the bivariate correlations of the questions were only weak, which would have limited the usefulness of imputing missing values based on the information of observed values. The advantage of the applied complete case analysis was therefore the independence from further assumptions, regarding a missing data generating process and an appropriate imputation method, and the consequently distinct data basis for analysis. Finally, it should be noted that new variants of the SARS-CoV-2 virus evolve rapidly, recently the delta variation. This makes it difficult to estimate how long the decision rules will continue to apply. In addition, the presentation of symptoms may vary between different countries^16^, which could be due to cultural habits, social conditions, or even genetics. However, we think that applicability can be expected at least for Europe, because the questionnaire consists of very basic health questions. Therefore, we suggest establishing sentinel practices for rapid detection of clinical patterns. This could foster the adaption of clinical decision rules on different variants during the pandemic waves in diverse countries.

To conclude, the combination of four key items allowed the exclusion of SARS-CoV-2 infection with high certainty in family practice. Depending on available resources, consideration could be given to omitting PCR testing in this group, which still represents more than one-third of the collective. This could be particularly relevant for countries where PCR testing is difficult to obtain or very expensive. With the goal of 100% exclusion of SARS-CoV-2 infection to prevent the spread of SARS-CoV-2 to the population level, COVID-PCR testing could only be saved in patients with negative response in all items. In addition, the decision rule might also help for ruling-in SARS-CoV-2 infection in terms of rapid assessment of infection risk. Future studies should investigate whether the combination of self-reported symptoms and simple contact history questions could also help to increase the diagnostic or prognostic power of the rapid antigen test at the population level, which is currently under discussion^17^.

## Supplementary information

Reporting Summary

## Data Availability

The data set analyzed during the current study is available from the corresponding author on reasonable request.
