# Supplementary material for: COVID-19 assessment in family practice—A clinical decision rule based on self-rated symptoms and contact history
Source: NPJ Prim Care Respir Med. 2021 Nov 25;31:46. doi: 10.1038/s41533-021-00258-4 (PMC8617029; doi:10.1038/s41533-021-00258-4)
Supplement: Supplementary file 1 — Reporting Summary [file 41533_2021_258_MOESM1_ESM.pdf]

nature portfolio

Corresponding author(s): Antonius Schneider

Last updated by author(s): Jul 26, 2021

Reporting Summary

Nature Portfolio wishes to improve the reproducibility of the work that we publish. This form provides structure for consistency and transparency in reporting. For further information on Nature Portfolio policies, see our Editorial Policies and the Editorial Policy Checklist.

Statistics

For all statistical analyses, confirm that the following items are present in the figure legend, table legend, main text, or Methods section.

n/a	Confirmed	
☐	☒	The exact sample size (n) for each experimental group/condition, given as a discrete number and unit of measurement
☐	☒	A statement on whether measurements were taken from distinct samples or whether the same sample was measured repeatedly
☐	☒	The statistical test(s) used AND whether they are one- or two-sided<br>*Only common tests should be described solely by name; describe more complex techniques in the Methods section.*
☐	☒	A description of all covariates tested
☐	☒	A description of any assumptions or corrections, such as tests of normality and adjustment for multiple comparisons
☐	☒	A full description of the statistical parameters including central tendency (e.g. means) or other basic estimates (e.g. regression coefficient) AND variation (e.g. standard deviation) or associated estimates of uncertainty (e.g. confidence intervals)
☒	☐	For null hypothesis testing, the test statistic (e.g. F, t, r) with confidence intervals, effect sizes, degrees of freedom and P value noted<br>*Give P values as exact values whenever suitable.*
☒	☐	For Bayesian analysis, information on the choice of priors and Markov chain Monte Carlo settings
☒	☐	For hierarchical and complex designs, identification of the appropriate level for tests and full reporting of outcomes
☒	☐	Estimates of effect sizes (e.g. Cohen's d, Pearson's r), indicating how they were calculated

Our web collection on statistics for biologists contains articles on many of the points above.

Software and code

Policy information about availability of computer code

Data collection	Collection of data with paper questionnaire.
Data analysis	Computations were conducted with R 4.0.3 (The R Foundation for Statistical Computing, Vienna, Austria).

For manuscripts utilizing custom algorithms or software that are central to the research but not yet described in published literature, software must be made available to editors and reviewers. We strongly encourage code deposition in a community repository (e.g. GitHub). See the Nature Portfolio guidelines for submitting code & software for further information.

Data

Policy information about availability of data

All manuscripts must include a data availability statement. This statement should provide the following information, where applicable:

- Accession codes, unique identifiers, or web links for publicly available datasets
- A description of any restrictions on data availability
- For clinical datasets or third party data, please ensure that the statement adheres to our policy

The data set analyzed during the current study is available from the corresponding author on reasonable request.

Field-specific reporting

Please select the one below that is the best fit for your research. If you are not sure, read the appropriate sections before making your selection.

☒ Life sciences ☐ Behavioural & social sciences ☐ Ecological, evolutionary & environmental sciences

For a reference copy of the document with all sections, see nature.com/documents/nr-reporting-summary-flat.pdf

Life sciences study design

All studies must disclose on these points even when the disclosure is negative.

Sample size	1141
Data exclusions	Questionnaire with incomplete data were excluded. Most of the missing values were observed in 'age' (5%), 'stay in corona risk area' (3%), 'contact with persons with suspected infection' (3%), 'chronic disease' (2%), 'sudden disease onset' (2%) and 'smoking' (2%). Other information was missing in less than 1% of the cases. In general, imputation is most helpful in a 'missing at random' (MAR) setting, which allows the derivation of unobserved values from the information given by the observed values of an observational unit. Given the aforementioned variables/features with missing values and the other variables/features of the questionnaire we did not assume meaningful relations, for example between 'smoking' and 'fever'. We also explored the bivariate (Spearman) correlation of variables and found weak correlations (r<0.3) in most of the cases (86%). We critically discussed these facts and finally decided to use a complete case analysis. This seems particularly reasonable given that we have achieved the intended sample size. It comes with the advantage of being able to work with a single and completely observed dataset, without relying on further assumptions about the missing data generating process and appropriate imputation procedure.
Replication	Replication was not possible with a diagnostic study design.
Randomization	Randomisation was not possible within this diagnostic study.
Blinding	The patients were blinded against the PCR results.

Reporting for specific materials, systems and methods

We require information from authors about some types of materials, experimental systems and methods used in many studies. Here, indicate whether each material, system or method listed is relevant to your study. If you are not sure if a list item applies to your research, read the appropriate section before selecting a response.

Materials & experimental systems

n/a	Involved in the study	
☒	☐	Antibodies
☒	☐	Eukaryotic cell lines
☒	☐	Palaeontology and archaeology
☒	☐	Animals and other organisms
☐	☒	Human research participants
☐	☒	Clinical data
☒	☐	Dual use research of concern

Methods

n/a	Involved in the study	
☒	☐	ChIP-seq
☒	☐	Flow cytometry
☒	☐	MRI-based neuroimaging

Human research participants

Policy information about studies involving human research participants

Population characteristics	All consecutive patients (at least 18 years old) who came for COVID-PCR were asked to complete a short questionnaire on medical history, self-reported symptoms and possible contact with (potentially) infected persons.
Recruitment	Patients were included consecutively.
Ethics oversight	The study was approved by the Ethics Committee of the Medical Faculty of the Technical University of Munich.

Note that full information on the approval of the study protocol must also be provided in the manuscript.

Clinical data

Policy information about clinical studies

All manuscripts should comply with the ICMJE guidelines for publication of clinical research and a completed CONSORT checklist must be included with all submissions.

Clinical trial registration	*Provide the trial registration number from ClinicalTrials.gov or an equivalent agency.*
Study protocol	*Note where the full trial protocol can be accessed OR if not available, explain why.*
Data collection	*Describe the settings and locales of data collection, noting the time periods of recruitment and data collection.*
Outcomes	*Describe how you pre-defined primary and secondary outcome measures and how you assessed these measures.*
